# Ranking environmental and edaphic attributes driving soil microbial community structure and activity with special attention to spatial and temporal scales

**DOI:** 10.1002/mlf2.12116

**Published:** 2024-03-26

**Authors:** Vadakattu V. S. R. Gupta, James M. Tiedje

**Affiliations:** ^1^ CSIRO Agriculture & Food Urrbrae South Australia Australia; ^2^ Centre for Microbial Ecology Michigan State University East Lansing Michigan USA

**Keywords:** abundance, community structure, diversity, functional microbiome, microorganisms

## Abstract

The incredibly complex soil microbial communities at small scales make their analysis and identification of reasons for the observed structures challenging. Microbial community structure is mainly a result of the inoculum (dispersal), the selective advantages of those organisms under the habitat‐based environmental attributes, and the ability of those colonizers to sustain themselves over time. Since soil is protective, and its microbial inhabitants have long adapted to varied soil conditions, significant portions of the soil microbial community structure are likely stable. Hence, a substantial portion of the community will not correlate to often measured soil attributes. We suggest that the drivers be ranked on the basis of their importance to the fundamental needs of the microbes: (i) those that supply energy, i.e., organic carbon and electron acceptors; (ii) environmental effectors or stressors, i.e., pH, salt, drought, and toxic chemicals; (iii) macro‐organism associations, i.e., plants and their seasonality, animals and their fecal matter, and soil fauna; and (iv) nutrients, in order, N, P, and probably of lesser importance, other micronutrients, and metals. The relevance of drivers also varies with spatial and time scales, for example, aggregate to field to regional, and persistent to dynamic populations to transcripts, and with the extent of phylogenetic difference, hence phenotypic differences in organismal groups. We present a summary matrix to provide guidance on which drivers are important for particular studies, with special emphasis on a wide range of spatial and temporal scales, and illustrate this with genomic and population (rRNA gene) data from selected studies.

## INTRODUCTION

Soil environment is complex both in terms of physical organization and chemical composition encompassing a diverse range of environments or microhabitats in terms of accessibility, redox potential, osmotic potential, moisture and temperature regimes, and carbon (C) and nutrient concentrations. The large diversity and complex microbiome structure in soil ecosystems, for example, 10^7^–10^9^/g soil representing thousands of taxa encompassing all three domains of life, are a key component of managed (agricultural) and native systems to maintain functional capacities related to C and nutrient cycling, plant health and productivity, and overall ecosystem health. While these microbiomes can respond to short‐term management and environmental factors, since soil provides a protective environment, and its microbial inhabitants have long adapted to varied soil conditions, significant portions of the soil microbial community structure are likely stable. One of the dilemmas in soil biology research is how much of the focus should be on the composition versus their functions. Recently, Flamholz and Newman^1^ suggested that “species doesn't matter, but their ‘metabolism counts’ or another way of saying this is that it's the ‘functions that pays the bills’.” Nonetheless, information about the catalysts, the microbes, underpins the understanding of function as many basic traits are conserved phylogenetically, but it is also clear that not all microbial relatives have the same functions or respond in the same way to environmental drivers.

One of the major organizing principles for understanding the large species diversity in soil is the availability of energy sources and electron acceptors, especially oxygen concentration. The complexity of soil microbiome is also dependent on the extent of dispersal of natural inoculum, the selective advantages of those organisms under the environmental attributes of the new habitat, and the ability of those colonizers to sustain themselves over time. Soil microbial community structure is driven by a diverse range of (i) soil factors related to providing a suitable habitat in terms of physicochemical attributes, (ii) environmental drivers that regulate habitat characteristics, and (iii) host (e.g., plant and fauna)‐related factors (Table 1).

**Table 1:** Soil attributes ranked according to importance in their influence on microbial communities and the associated metadata requirements suggested by four soil microbiome guidance initiatives.

Attributes	Property	Measurement	AgriMicrobiome	Aust Microbiome	Earth Microbiome	GSC
Energy	OC quality/quantity	OC/TC	OC/TC	OC/TC	OC/TC	OC
	Light	Depth unless understory	Depth	Depth	Depth	Depth
Electron acceptor	O_2_	Porosity, moisture, color	Optional, BD, moisture	N/A	Optional, BD	Moisture, BD, drainage characteristics
	Fe^3+^/SO_4_^2−^/NO_3_^−^	Available nutrients	NO_3_, S	NO_3_	NO_3_	TN, ON
Environmental effectors	pH	pH	pH	pH	pH	pH
	Salt	EC	EC	EC	EC	EC
	Texture	Texture	Texture	Texture	Texture	Texture
	Drought/moisture‐rainfall	MAP	Inferred	Rainfall	Inferred	Rainfall
	Temp	MAT	Inferred	MAT	MAT	Temp
	Toxic chemicals	Situation dependent	Exc. Al	Exc. Al	Exc. Al	Heavy metals, agrochemicals
Host	Plants & seasonality	Plant type	Time of sampling, plant type	Time of sampling, plant type	Time of sampling, plant type	Time of sampling, plant type
	Animals & manures	Situation dependent	Situation dependent	Situation dependent	Situation dependent	Situation dependent
	Fauna	Situation dependent	Situation dependent	Situation dependent	Situation dependent	Situation dependent
Nutrients	N	Available N	NO_3_ or NH_4_	NO_3_ or NH_4_	NO_3_ or NH_4_	TN, ON
	P	Available P	Optional	Optional	Optional	P
	Micronutrient	Situation dependent	Optional	Optional	Optional	Optional

Measurements such as those related to concentrations of toxic chemicals, micronutrients, application of organic manures, and host are situation‐dependent.

AgriMicrobiome, Agricultural Microbiome Project^2^; Aust Microbiome, Australian Microbiome Project^3^; BD, bulk density; C, carbon; Earth Microbiome, Earth Microbiome Project^4^; EC, electrical conductivity; Exc. AI, exchangeable aluminium; GSC, Genomic Standards Consortium^5^; MAP, mean annual precipitation; MAT, mean annual temperature; N, nitrogen; OC, organic carbon; ON, organic nitrogen; P, phosphorous; TC, total carbon; Temp, temperature; TN, total nitrogen.

At larger spatial scales, for example, at a field, landscape, regional, or latitudinal scale, factors that influence soil formation such as parent material, climate, vegetation, topography, and the time for weathering result in variations in soil type and physicochemical properties and those different soil environments affect microbial composition and functions. Whereas at smaller spatial scales, distinct soil environments may only be separated by micrometers to centimeters but vary in terms of organic matter quantity and quality (dissolved and particulate), pH, pore geometry and accessibility, aggregate size and stability, recent disturbance, fine roots, etc., and hence, in microbial abundance, community composition and activity^6^, ^7^, ^8^, ^9^. Additionally, within a location (soil profile), soil habitat conditions usually vary with depth and by the influence of plants and mesofauna and macrofauna, which, through selective predation, significantly affect microbial composition and abundance. Ability to predict a future scenario of microbial community structure and functions in a changing environment, purposeful or incidental, for example, climate, is one of the major goals of microbial ecology research. However, as noted above, many of the soil communities are often not correlated to measured soil attributes, and appear to be assembled by chance, i.e., stochastic as opposed to deterministic. Thus, the complexity of independent and inter‐related factors that impact the soil microbiome results in a disconnect between a large‐scale impact (e.g., temperature) and the microbial community. Zhou and Ning^10^ have summarized features and analyses of stochastic community assembly processes that pose a major challenge to predictive microbial ecology research as they make the community structure and its functions less or even unpredictable. Some of the possible reasons for this mismatch are as follows: (i) difference in the time of measurement that may not be directly relevant for the community structure at that specific time; (ii) the level of resolution in the community description is not sensitive enough or appropriate to show differences in community structure; and (iii) the level of sensitivity of the specific metric or attribute measured to detect change, etc. Additionally, space and time are broad, and when or where measured may not be appropriate for the reported data, hence a mismatch.

Previous discussion on the variability in microbial communities in terrestrial ecosystems is generally based on correlations between community diversity and composition and the soil abiotic and biotic (aboveground/host) factors, with limited explanation on the mechanistic factors that can influence microbial growth and function. The importance of various factors identified has been based on the ability to detect their effects and the ease of measurement of different properties^2^, ^3^, ^4^, ^5^, ^11^, ^12^. Recently, Fierer^13^ categorized biotic and abiotic factors based on their relative importance in structuring soil bacterial communities based on what is known about their spatial patterns considering that the factors are often not independent and can correlate with one another. Although, during the last two decades, a number of studies have discussed some aspects of the relationships between edaphic and environmental factors on biogeography or variation in microbial communities, no systematic analysis of drivers/factors has been carried out to identify what governs community composition, diversity, etc., especially based on physiological requirements of organisms, ecological understanding of various organisms, particularly for different functional categories, and considering spatial and temporal scales. In addition, as a large proportion of the soil microbial community is inactive and thus may not respond quickly (or at all) to environmental changes can confound conclusions based on alterations in the overall community structure. In natural terrestrial systems, spatial variation at the local (e.g., aggregate and plot) scale involves differences in soil structure, water, C, and nutrients, whereas at larger (field, landscape, regional, etc.) scales, variations in soil type, topography, and vegetation composition can also play significant roles. Some natural disturbances such as fire and floods can cause major changes in resource availability and the physical environment affecting microbial composition, but most natural environmental changes are slow and/or subtle, thus not causing major changes in microbial communities^14^, ^15^, ^16^. Changes in land use history induced by humans including land use intensification and management practices introduced to improve agricultural productivity have often been associated with changes in taxonomic and functional microbial biodiversity.

Sampling volumes for high‐complexity environments like soil often fail to adequately capture microbial diversity, owing to microscale heterogeneity and bottleneck effects during population subsampling that lead to the stochastic proliferation of some taxa and the loss of others^17^, ^18^. Artifacts from random sampling in microbial ecology studies using sequencing‐based technologies can greatly overestimate β‐diversity, that is, the “under sampling problem”^19^, which is best addressed by increasing the number of technical and biological sample replicates. Sample size has been reported to significantly influence bacterial and fungal community structure results with significant effects on α‐ and β‐diversity of communities^18^. In the case of bacterial communities, while dominant members may mask the signatures of minority populations, i.e., rare biosphere, sufficiently deep amplicon sequencing can overcome these limitations by revealing minority populations in the context of the overall community structure^18^. Unlike the bacteria, patchiness and spatial clustering are often associated with soil fungi, i.e., locally abundant but spatially rare phylotypes, due to their preferential association of localized spore structures with decomposing crop residues; thus a larger sample size may assit with detection of low‐abundance taxa^20^, ^21^. Additionally, pooling strategies that are generally used in many sequencing‐based studies may reduce sample numbers and variability, but they could mask a significant portion of fungal communities affecting richness‐ and structure‐related indices, via community homogenization^21^. This would also apply to spatially distributed bacterial and protistan communities and functional populations (e.g., diazotrophs and denitrifiers) exhibiting patchiness at local scales for which pooling may mask connections with edaphic factors.

This concept paper briefly discusses what is known from the literature and aims to organize this information based on the first principles (Rules of Life; RoL) of what is essential for microbial growth in terms of energy, what are the major effectors that often limit growth, what are the macroorganisms and their influences, and finally what are the other nutrients or physicochemical influences of their soil “home”. While it is key to identify what is important to measure to describe the microbes' habitat, it is also important to consider what attributes can reasonably be measured, and if they cannot, what is a reasonable substitute that will provide at least some or most of the information for that attribute (Table 1).

## WHAT ARE THE ENVIRONMENTAL DRIVERS: WHAT IS CURRENTLY PROVIDED IN METADATABASES?

### Examples of metadata databases

Attempts to correlate soil microbial community structures with their environment require substantive, good‐quality, and consistent soil habitat data. During the last decade, a number of soil environmental metadata databases have been suggested and established by various organizational, national, and international consortia, for example, Genome OnLine Database (GOLD)^11^, Genomic Standards Consortium (GSC)^5^, Earth Microbiome project (https://earthmicrobiome.org/)^4^, the National Center for Biotechnology Information's BioSample Database (https://www.ncbi.nlm.nih.gov/biosample), Australian Microbiome (https://www.australianmicrobiome.com/)^3^, and AgriMicrobiome^2^  (Table 1). For the human microbiome‐related research, the International Human Microbiome Standards (IHMS; https://human-microbiome.org/) promoted the development and implementation of standard operating procedures for sample identification, collection, processing, and data quality optimization for improved comparability. However, in terrestrially related research, the multiple databases required or collated by individual researchers, national, and international consortia present a plethora of combinations that are mostly determined or collated based on individually observed and specific topics of interest. The Terragenome International Soil Metagenome Sequencing Consortium was the first to establish standards and data types for describing soil features relevant to soil biology research^22^ and later the soil biology research community evaluated the importance and likelihood of those being actually measured because of their complexity or cost, and if not a high‐value trait, a substitute is recommended that would yield similar trait information^23^, this then became the current soil standard in the GSC. The GSC now reports this as minimum information standards, i.e., Minimum Information about any Sequence (MIxS) environmental packages in terms of environment and host‐associated contextual data specific to distinct environments. For soil, this is MIxS‐soil and for plants, this is MIxS‐plant. These were later endorsed by the Earth Microbiome Project^4^. Other notable metadata standards and lists include the Australian Microbiome Project^3^ and the AgMicrobiomes Research Coordination Network (RCN)^2^. Many of the data sets have checklists of required or essential and optional (desirable) metadata standards, with each group suggesting the minimum information to interrogate the genomic data to identify factors modulating variations in microbiomes. Dundore‐Arias et al.^2^ proposed the need to follow or maintain community‐driven metadata standards for agricultural research in terms of properties that are “required” and “desirable” and ontologies essential for inclusion in a minimum metadata list and discussed the need to include well‐annotated metadata for agricultural microbiomes. Other factors considered in preparing the checklists include costs and effort involved in collecting the information; for example, measurements requiring complex analysis were ignored or replaced with simple measurements. Overall, many factors are included based on known or assumed relationships, some due to ease of analysis; for example, various factors, including soil properties (e.g., pH), climate, vegetation type, and nutrient availability, have been shown to structure the composition of soil bacterial and fungal communities worldwide.

### Environmental drivers reflected in biogeography of microbes

A large body of research supports the idea that free‐living microbial taxa show biogeographic patterns^6^, ^8^, ^24^, ^25^, ^26^. Fierer and Jackson^6^ suggested that microbial biogeography is controlled primarily by edaphic variables and differs fundamentally from the biogeography of “macro” organisms. Martiny et al.^27^, in their review, confirmed the Baas–Becking hypothesis, that is, “the environment selects” and is responsible for spatial variation in microbial diversity (assuming no dispersal limitation). Hanson et al.^28^ suggested that processes such as selection, drift, dispersal, and mutation can create and maintain microbial biogeographic patterns on inseparable ecological and evolutionary scales. Malard and Guisan^9^ proposed a metabolic niche framework extending the previously used concept of a realized environmental niche to predict the geographic space distribution of species and suggested that it is possible to define metabolic niches of microorganisms including bacteria, fungi, and protists using multiomics‐based analysis of their genomic content. Goberna et al.^29^ reported that phylogenetic clustering in soil bacterial communities was stronger in habitats dominated by biotic filtering, and traits related to abiotic stress tolerance (e.g., desiccation) and competition (e.g., organic C use and ability to form nutrient‐scavenging structures) underlie phylogenetic clustering.

In a recent study using soil bacterial composition data for 1381 samples from across the Australian continent, Bissett et al.^30^ modeled the relative abundances of soil bacterial taxonomic groups to describe bacterial niche space and optima and suggested that “predictions of niche space and optima were dependent on taxonomic level and were not always congruent through subtaxa or with Structural Equation Model (SEM) path coefficients.” They report successful prediction of soil bacterial response through explanatory SEM properties developed using edaphic, climate, and spatial variables as indicators of microbial traits. This in turn would help predict potential ecosystem service response to near‐term expected changes in the Australian climate. However, at present, it is not clear if biotic and abiotic filtering mechanisms interact with phylogenetic conservation of niche preferences, resulting in distinct patterns of phylogenetically conserved spatial distributions.

In terrestrial systems, it has been broadly reported that microbial communities are structured at several spatial scales, suggesting the impact of abiotic conditions and land use, i.e., due to the spatial heterogeneity of soil properties; hence, micro‐scale heterogeneity is key to the coexistence of microbes from diverse phylogenetic groups and metabolic capabilities^7^, ^31^, ^32^. Also, co‐occurrence of large diversity could be attributed to partitioning of resources in space and time and/or trade‐offs between species–species interactions, particularly at smaller spatial scales. Delgado‐Baquerizo et al.^33^ reported that despite the overwhelming diversity of bacterial communities, relatively few bacterial taxa are abundant in soils globally. While this is true at the genus and above taxonomic levels, at a finer taxonomic scale, strong local endemicity is observed, for example, fluorescent *Pseudomonas* genotypes are not globally mixed^34^. Furthermore, the taxonomic resolution of microbial groups at finer spatial resolution, such as the millimeters to centimeters scale, could help determine the importance of species density, composition, and growth stage of plants in shaping microbial community composition and spatial patterns^35^.

Soil redox conditions are a dominant force in shaping microbial communities in the landscape^36^ since aerobic respiration and fermentation provide very different amounts of energy and select very different taxa. Topographic position, texture, drainage, depth to water table, and available carbon are key soil drivers of redox status. As one example, higher levels of oxygen and other electron acceptors in the surface layers allowed for greater diversity of microbes, while at depth, reducing conditions, reduced the community to a simpler core of anaerobes, dominated by fermenters (*Bacteroidota* and *Firmicutes* [*Bacillota*])^36^. Similarly, strong gradients in redox conditions along with the quantity and quality of C created by the rhizoplane–rhizosphere–bulk soil continuum combined with the small spatial scales induce selection at finer taxonomic resolution^37^. Recently, Fierer^13^ proposed a hierarchy and relative importance in terms of biotic and abiotic factors that can influence the structure of bacterial communities across space and time. He pointed out that these factors are not necessarily independent and can correlate with one another (e.g., soil moisture availability influenced by texture and aggregate composition). The suggested hierarchy was based on reports that evaluated spatial patterns in soil microbial communities^13^.

Unlike the soil bacterial community that is naturally spatially bound and depends on abiotic processes such as drift and dispersal, distribution of soil fungi at smaller spatial scales is influenced by their ability to form hyphal networks extending from centimeter to meter distances. At larger spatial scales, that is, global scale, climatic factors, followed by edaphic and spatial variables, are the best predictors of fungal richness and community composition^24^. Also, the plant‐to‐fungus richness ratio declines exponentially toward the poles. Maynard et al.^38^ reported linkages among functional trait expression, climate, and phylogeny of fungi in terms of abiotic stress tolerance related to moisture and thermal niches, suggesting the importance of relevant factors when looking at fungal trait expression across broader spatial scales. Additionally, unlike bacterial communities, host association may buffer the effects of edaphic and environmental/climate factors on fungal community composition, as host‐associated fungal communities (e.g., mycorrhizae) are more similar to each other across climatically distinct sites than the whole fungal community^39^.

Most of the research on the edaphic and environmental properties influencing microbial composition is related to the spatial‐scale variations from local to global scales by measuring soil or site characteristics. This may be because the temporal variation is generally considered as smaller than spatial variation^13^, ^40^; however, it is important to gain knowledge of factors driving temporal variability in terms of the speed and nature of response of microbiomes including their resilience to management and environmental changes. When comparing microbial community differences across land types, Lauber et al.^41^ reported that the numbers of taxa or lineages and α‐diversity were significantly influenced by the time of sampling, with the temporal variability in α‐diversity exceeding the variability between land‐use types. The temporal variability was significantly related to moisture and temperature. Differential responses to changes in temperature, moisture, and resource availability between different individual taxa or groups, for example, copiotrophs versus oligotrophs, contribute to temporal variations in the phylogenetic composition and functional activity of microbiomes^37^, ^42^. For example, microbial response patterns to initiation of favorable soil conditions (i.e., moisture status) display phylogenetic clustering and are primarily conserved at the subphylum levels, suggesting that it is a phylogenetically conserved trait^42^.

## ASSESSING THE KNOWLEDGE FROM CORRELATIVE RELATIONSHIPS AS DRIVERS OF MICROBIAL METABOLIC AND GROWTH REQUIREMENTS

Although most omics‐based microbial community studies present data on a list of habitat‐based properties, there is no systematic collection of information known or suggested to influence the growth and/or activity of microbial communities. Although there have been many studies recently presenting information on potential factors driving variations in microbial composition and/or diversity from correlative relationship‐based investigations, sometimes, it is unclear which specific factors or combination of factors most likely drove the observed spatial or temporal variations in the composition of soil microbiome. One reason is that randomly selected soil and environmental characteristics generally seem to explain only a small part of the total variation and another reason is that follow‐up tests of the relationship are often not conducted or are too difficult to conduct. Multivariate analysis approaches are generally used to determine the factors that drive variation in microbial community abundance and composition. However, due to the compounding effects from unmeasured environmental factors, it is almost impossible to measure all environmental variables in practice. As a result, for instance, the distance effect is most likely overestimated since it cannot exclude the impacts of unmeasured environmental variables^13^, ^19^.

The unexplained variation has also been attributed to lack of measurement of all the appropriate properties/factors either because of difficulties with accurate measurements or complex methodologies, especially microscale spatial or temporal variations. For example, while total soil organic C is generally considered as a suitable measure of energy sources, it is difficult to measure short‐term changes in biologically available C from root exudates in rhizosphere samples, but these are likely more relevant. Similarly, measurement of total concentrations of major and micronutrients is a common practice, although it is the biologically available forms that actually impact the abundance, composition, and activity. It is likely that smaller variations between such factors and populations may predominate, but our inability to determine the linkages at the smallest scale leads to the dilution of what otherwise may be a strong local correlation. However, our sampling of larger volumes results in weaker global correlations. Hence, a careful consideration of these factors is required, especially to adequately address/explain temporal variations in microbial composition and functions.

What is missing is a conceptual framework based on what is required for microbes to survive, grow, and function in terms of habitat properties and environmental conditions considering variations and changes both at spatial and temporal scales, i.e., similar to macroorganisms, variations in niche preferences for microorganisms may drive, in part, their spatial patterns. Understanding the niche preferences of microbial taxa is also important to predict how taxa or communities may respond to environmental changes. For example, Malard and Guisan^9^ found that edaphic properties were the most important drivers of both community diversity and composition for all microbial groups based on the estimation of the environmental niche breadth and position of each taxon. They reported that fungi showed the smallest niche breadth and bacteria and protist the largest niche breadth, followed by archaea. This information is necessary to identify patterns in microbial community diversity in order to determine what drives their complexity, and how and if complexity is important for functional activity and resilience.

Additional knowledge is required to address challenges to gaining a mechanistic understanding of the drivers and responses of microbial communities, their functions, and overall ecosystem dynamics across large, diverse geographic scales. Measurements of fluxes or flows of materials, energy, or information are needed across different levels of biological organization or spatial units. For example, microbes that are involved in the production and consumption of greenhouse gasses, that is, denitrifying microbes, operate and function at the submicrometer scale; however, the consequences of their collective contribution to green house gas (GHG) emission and climate change are observed at larger scales^43^. Hence, a dynamic theoretical framework and mathematical techniques need to develop quantifiable relationships to associate spatial, temporal, and phylogenetic factors with the microbial scale and community assembly. This is because microbial community assembly is a product of deterministic and stochastic processes with different mechanisms dominating at different scales^43^. Another example reported by Neal et al.^44^ demonstrates how soil management practices result in the emergence of distinct associations between biological functions and physical structure, i.e., “contrasting emergent soil structures exert selective pressures on the microbial metagenome” with effects on the C flux in the system and overall changes in soil organic C^44^. These examples suggest that for a detailed understanding of interactions between microbial communities and their drivers, it is important to consider mechanistic relationships between microbes and edaphic and environmental drivers.

**Figure 1:**
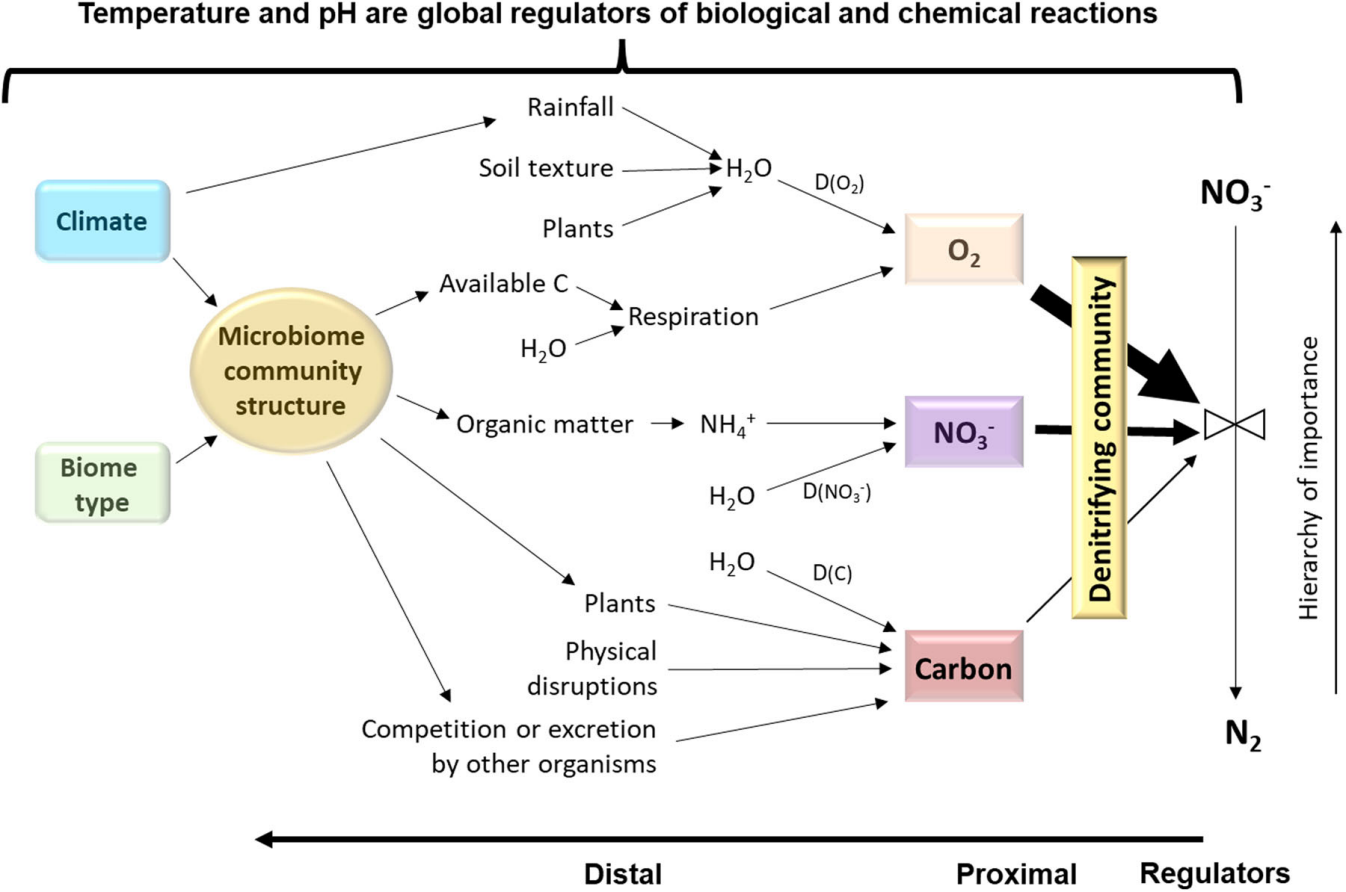
Relationships between different proximal and distal controlling factors of microorganisms involved in denitrification and the process (adapted from Groffman et al.^45^). While the different soil, plant, and environmental factors can be grouped into distal or proximal categories, temperature and pH effects are considered global across multiple factors.

Additionally, when studying factors that control a microbial process at different temporal and spatial scales, it is important to consider both primary or proximal factors that exert fundamental control and distal factors that control the dynamics of proximal factors (Figure 1)^45^. For example, while oxygen, nitrate nitrogen (N), and C are considered as the primary factors that control denitrification at the cellular level, they are influenced by many biological, physical, and environmental factors that could become more important, especially at higher spatial and temporal scales, and when quantifying the significance of the process at the scale required^45^. While the general microbial community structure as part of distal (biological) factors can affect the three edaphic proximal factors, denitrifying community composition is considered as another important primary (proximal) factor controlling the function^46^, ^47^.

## ATTRIBUTES/ENVIRONMENTAL VARIABLES–WITH PERCEIVED MECHANISTIC LINKS TO COMMUNITY STRUCTURE

### Energy

Electron‐transfer reactions are fundamental to energy production in all organisms including organisms that are autotrophic or heterotrophic, free living or an obligate parasite; every cell must solve the energy‐conservation problem to survive, hence the need for both an electron donor, often an organic C source, and an electron acceptor, often oxygen, but for some bacteria, it can be nitrate, sulfate, ferric iron (which are also “nutrients”), and some chlorinated compounds.

#### Electron donors

**Figure 2:**
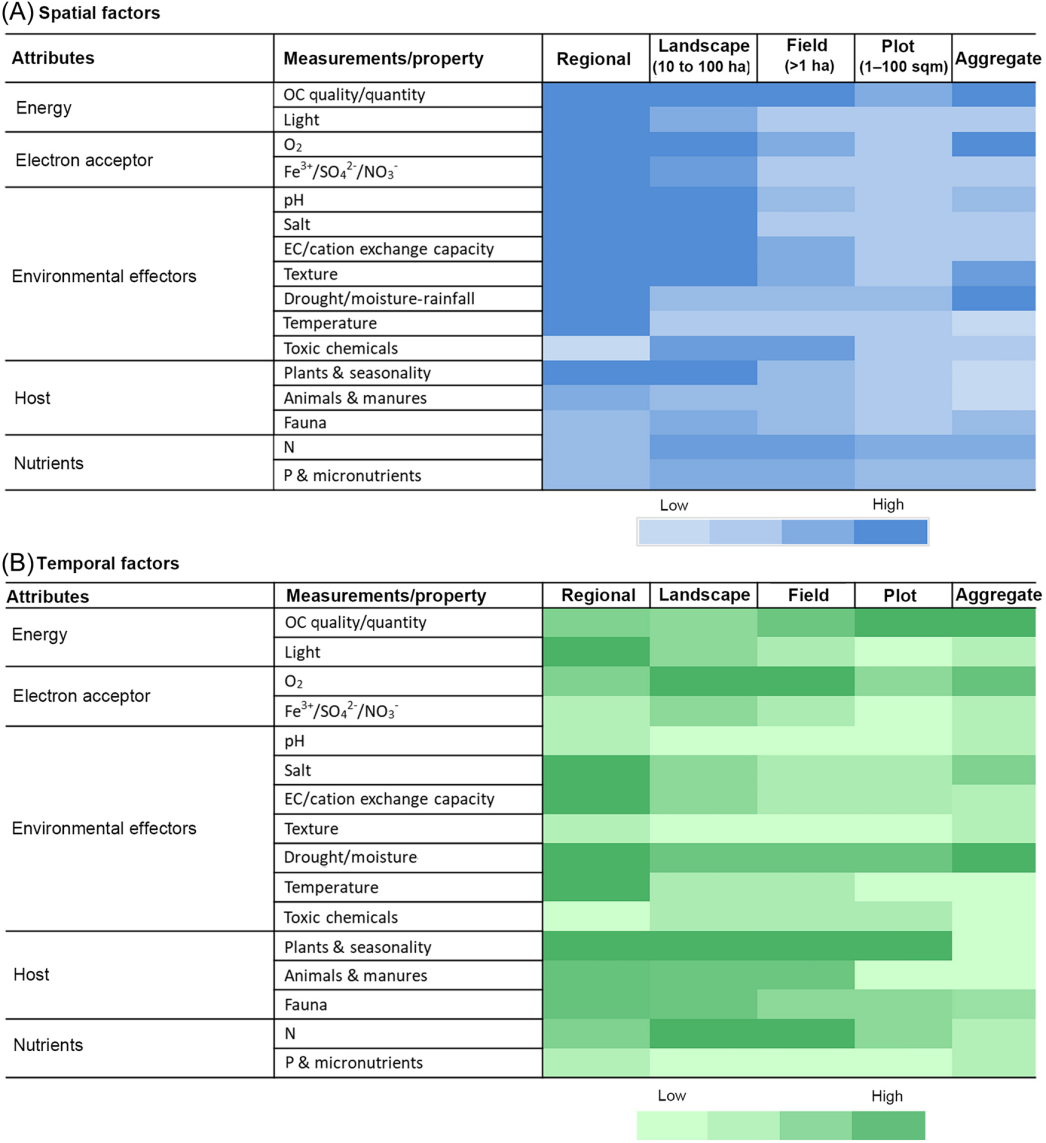
Relative importance‐based ranking of spatial and temporal factors influencing microbial community composition and diversity in soil systems. (A) Spatial factors. (B) Temporal factors. The color scale indicates the relative importance of the measure at the indicated scale.

Soil organic C is most strongly related to microbial abundance and composition and the relationship has been recognized to be important both spatially and temporally^25^, ^33^, ^48^, ^49^. Different soil environments vary in the energy resources that they provide depending upon the sources of C inputs, that is, nature of detritus from plants. Although plants are the main contributors of C from their aboveground and belowground biomass, the quality of plant C inputs and the varying decomposition rates of different plant residues may result in a weaker relationship between net plant productivity and microbial composition. Community composition will also be determined by the succession of species since they influence the quality and quantity of resources available, for example, early degraders of easily degradable compounds and/or incompletely metabolized products left behind from the breakdown of complex macromolecules^50^, ^51^. Also, due to their smaller size, microorganisms, in particular bacteria and archaea, could partition more and diverse niches at finer scales, that is, millimeters to centimeters, and hence be influenced by the spatial heterogeneity in the availability of energy sources, in particular temporal variability. This highlights the need to document temporal changes in the quantity and/or quality of biologically available C to better relate to the composition and ecology of a microbial community^13^, ^52^ (Figure 2). In addition, in most soil environments, especially in agricultural soils, boom and bust environments may create discrete niches due to resource concentrations varying in time, for example, rhizosphere soils in‐crop versus noncrop situations, and the detritus microbiome may determine the metagenome of developing root systems i.e., legacy effects^53^, ^54^.

Differences in the spatial distribution and temporal variation of the quantity and quality of organic C sources from the land‐use change from natural to agricultural fields contribute to variations in microbial diversity and composition including various functional microbial groups. The rapidly fluctuating soil environments with highly variable resource gradients (e.g., soluble or particular organic C pool) in the agricultural soils provide a wide range of niches for microbial growth, resulting in distinct differences in microbial community structure compared to stable natural systems^55^. In contrast, due to the lack of disturbance in natural systems, the habitat heterogeneity of C sources would make a significant contribution to variations in microbial communities at a smaller scale, for example, aggregate, plot, and depth, compared to agricultural systems. While microbial communities may be limited by nutrient availability, fertilizer addition in cropped fields may result in changes in specific groups of microbes (e.g., autotrophic microbes involved in nitrification, denitrification, and sulfur (S)‐oxidation in response to fertilizer N or S addition), shifts in r‐ to k‐selected species, and even colonization by new species from global/regional pools, thereby affecting spatial variation in microbial communities^55^, ^56^, ^57^.

#### Agricultural systems—An important case

Tillage and other physical disturbance practices and conversely conservation tillage (i.e., no tillage) can cause significant alterations to multiple attributes related to C resource availability (e.g., quantity, quality, and duration of availability), soil habitat structure, physiobiological environment, etc., thereby causing significant shifts in bacterial and fungal community and diversity including the ratio of bacteria to fungi^58^, ^59^, ^60^. The influence of such factors was shown to extend from an aggregate level (microsite level) to a larger spatial scale (e.g., field to landscape levels), exerting a significant influence on the structural composition, i.e., diversity and relative abundance of various phylogenetic and physiological groups (e.g., copiotrophs and oligotrophs) and functional potential of microbial communities, although the nature and magnitude of effect may vary between different aggregate size classes and based on soil type^61^, ^62^. As tillage is part of a suite of management practices, such as crop rotation, and fertilizer and agrochemical use, its effect can vary across different temporal scales depending upon differences in energy availability‐related factors^41^, ^55^, ^60^ (Figure 2). Primary productivity in agricultural systems is a critical factor modulating the quantity and quality of C resources and, therefore, one of the key drivers of microbial diversity and composition. Sunlight, moisture, and temperature, however, can be considered as surrogates for primary productivity. Similarly, decomposition is an important process with a significant influence on C availability, and this process is also influenced by temperature and moisture.

#### Microorganisms at depth

While most of the research on the composition and responses in soil microbial communities has focused on the surface (0–15 cm) soils, subsurface soils also harbor a diverse and active microbial community. These deep soil microbial communities play an important role in soil‐forming processes and more likely contribute to long‐term C sequestration as they are enriched with microbially derived C compounds (necromass) and characterized by high mean residence time^63^, ^64^, ^65^. The abundance of bacteria and fungi and α‐ diversity generally decreases with depth, especially for fungi, but archaeal diversity increases with soil depth^65^, ^66^, ^67^. The effects of soil depth on microbial/bacterial and fungal community diversity and composition have been shown to be related to variations in edaphic factors including organic C, changes in soil moisture, aerobic and anaerobic conditions (e.g., oxygen concentration/redox potential), bulk density, etc.^65^, ^67^, ^68^. For example, surface soils generally contain higher soil organic matter (SOM) and nutrient availability and harbor higher diversity of microorganisms; hence, richness and diversity in microbial communities decrease with increasing soil depth^68^, ^69^. Also, microbial communities in the surface soils are influenced by plant type‐based selective enrichment from differing root exudates, rooting pattern, residue decomposability, and faster‐changing environmental conditions, all of which result in more dynamic temporal variations in community composition.

Microbial communities in surface soils are more strongly influenced by management‐based (e.g., tillage, agrochemicals, and fertilizers) and environmental (e.g., temperature and moisture) disturbances compared to those at depth, which leads to limited short‐ and medium‐term changes in the abundance and composition. Hence, Banerjee et al.^65^ found stronger soil depth‐based variations in microbial diversity and community structure than cropping patterns in agricultural fields in southeastern Australia; however, a core group of operational taxonomic units (OTUs) comprising keystone taxa was found across soil depth. Although network complexity in bacterial and fungal communities generally seems to decline with depth, the relative abundance of all keystone OTUs increased with depth^65^. Even though network complexity declined, the contrasting patterns in some classes within the bacterial phyla *Acidobacteriota*, *Verrucomicrobiota*, and *Firmicutes* (*Bacillota*) with depth in bacterial communities associated with biofuel cropping systems may be related to the different ecological specialization^69^. For example, the relative abundance of many members of *Chloroflexi* (*Chlorofexota*) and *Nitrospirae* (*Nitrospirota*), considered as oligotrophic, increased with increasing depth.

#### Electron acceptors

The availability and concentration of electron acceptors such as oxygen and inorganic molecules such as NO_3_^−^, SO_4_^2−^, Fe^3+^, Mn^4+^, and SeO_4_^2−^ can support energy production in the absence of oxygen through microbial respiration in microbes with those physiologies. Aerobic respiration produces much more usable energy in the form of ATP (oxidative phosphorylation) than anaerobic pathways (fermentative, i.e., substate level phosphorylation). Thus, soil habitat characteristics that modulate the availability of electron acceptors play a significant role in determining the composition of microbial communities. Many soil habitats are characterized by a mixture of aerobic and anaerobic microsites, with their spatial distribution determined by their texture, aggregate, and pore structures and modulated by management practices and the nature of the ecosystem (disturbed agricultural fields vs. undisturbed native systems). These microsites can vary in terms of their oxygen concentrations due to environmental factors (e.g., rainfall) and soil physical properties (e.g., bulk density, drainage/infiltration properties, and soil texture), resulting in varying periods of anaerobic, microaerophilic, and anaerobic conditions that are variable both spatially and temporally. Additionally, the rate of consumption of O_2_ by microbes is faster than the rate of diffusion in soil environments; thus, microscale distribution of different microbial species is influenced by their ability to cope with changing oxygen concentrations^70^. Thus, oxygen concentration at any time is a product of supply and demand controlled by many factors, including those related to the soil matrix, physicochemical properties, plant and microbial actions, and environment, and should thus be considered at different scales both spatially and temporally (Figure 2).

Unlike the prokaryotic microorganisms (bacteria and archaea), fungi can traverse through soil pore networks through their hyphal networks and may be exposed to varying oxygen concentrations^44^, ^61^, ^71^. While the general assumption of aerobic heterotrophic respiration and the microorganisms capable of related metabolic processes as dominant groups in aerated soils, anaerobic microsites and switching of conditions from aerobic to anaerobic conditions can shift microbial metabolism from aerobic to less efficient fermentation. All of these can have a significant effect on the composition of microbial communities in soil ecosystems.

Soil textural and structural properties have a significant influence on the spatial and temporal variations in O_2_ concentrations. Physical properties of soils such as soil texture, i.e., relative proportions of clay, silt, and sand particles, which provide the framework for soil habitat structure, have been considered as important factors that govern the degree of microscale heterogeneity in terms of O_2_ concentrations through distribution of pore sizes, morphologies, distribution, and location of nutrients and other chemical species from their absorption to mineral particles (soil texture examples^13^, ^32^). Here, we discuss the textural and structural properties mainly in terms of their influence on O_2_ as an important electron acceptor and the influence of other soil‐related properties such as organic matter availability. Cation exchange capacity (CEC) is discussed in other sections. Variations in the soil texture and pore structure of soils can affect water availability and water and resource transport in addition to the movement of microbes, especially bacteria, fungal spores, and Protista^72^, ^73^, ^74^.

Soil textural‐based differences in microbial composition are mainly considered important in terms of variations in spatial patterns in microbial communities. The effect of soil texture variations on microbial diversity, composition, and functionality is considered greater at larger spatial scales (e.g., regional and landscape levels) due to differences in soil structure‐associated physical properties including water, C, and nutrient availability along with variations in vegetation type and biomass inputs. However, at smaller spatial scales, microscale heterogeneity in habitat conditions and resource availability are considered as the foundations of the coexistence of diverse microbes and high microbial diversity in soil environments. There is considerable evidence for spatial variability in the structure of soil microbiome and distribution of specific microbial groups of both bacterial and fungal communities in different size structural units (e.g., aggregates), pore structures, and particle sizes^32^, ^75^. For example, the relative abundances of bacterial groups both at phyla and sublevel taxa are affected by soil texture; i.e., relationships of different bacterial taxa with clay vs. sand are generally opposite as concentrations of sand and clay are negatively correlated^32^, ^48^, ^49^. With fungal taxa, the relative abundances of most dominant groups such as *Ascomycota* and *Basidiomycota* seem to show a positive relationship with silt/clay and a negative relationship with sand; however, responses in terms of individual fungal genera/species may depend upon their ability to establish hyphal networks that will enable them to access the complex pore structure, which varies with soil texture variation^44^.

Pore size distribution also plays a role in niche differentiation, competitive exclusion, and physical exclusion based on size for some groups of microorganisms. Additionally, plant rhizosphere legacy effects in terms of intra‐aggregate pore structure and microbial community composition may last in soil for 4–9 months, suggesting that soil texture‐ and structure‐based effects may have some temporal significance^75^. For example, water film fragmentation and intermixing of water‐ and air‐filled porosity distribution within macropores have been linked to greater bacterial species richness in coarser‐textured soils and provided no limitations in C availability^76^. The effects of soil texture and structure on temporal variations in microbial community structure are considered smaller and are mainly linked to habitat‐associated properties like soil moisture, air–water balance (aerobic and anaerobic spaces/conditions), and transport processes. For example, in soils with higher clay content and/or poorer aggregate and pore structure, reducing water infiltration and drainage resulted in water‐saturated conditions for longer periods. Therefore, soil physical properties related to texture and structure can play a significant role in determining the overall community composition and diversity, including microbial groups with specific physiological requirements and their metabolic capabilities. While the habitat conditions related to air–water balance can vary at short time scales, the overall soil structure can determine the long‐term habitat status; hence, these properties are considered important at different spatial and temporal scales (Figure 2).

### Environmental effectors/stressors

The survival and growth of microorganisms in soils are often influenced by persistent abiotic stressors such as low availability of water, acidic/alkaline conditions, high salinity, anoxia due to long‐term flooding, starvation, toxic metals or chemicals, and long‐term excessive heat, which are especially important at present due to climate change. Frequent occurrence of such disturbances will modify the habitat conditions, including the distribution of resources across space and time. Certain environmental conditions have a larger effect on biology, which is also true for soil microbes. We term these stressors as they are often beyond the normal physiological range, but for some microbes, they may be optimum and hence they select for those types of microbes. Also, these factors will influence the distribution of specific microbes or groups that can adapt in the short and long term to changes in conditions. These conditions, however, will generally reduce diversity, and as such are next to energy in importance as drivers of the soil community.

#### Soil pH and chemical environment

Soil environmental conditions related to chemical characteristics can vary both spatially and temporally, potentially affecting the survival, growth, and activity of microorganisms. For more than a century, soil pH has been known to be one of the primary soil parameters driving the composition of the microbial community and its processes at both global and local scales, although different processes appear to be influencing the community assembly of bacteria and fungi^3^, ^13^, ^24^, ^48^, ^49^, ^77^. For example, soil pH generally explains a significant proportion of the variability associated with the composition and abundances of bacterial communities and the phylogenetic structure of total bacterial communities within each dominant lineage; i.e., the effect of soil pH on the composition of bacterial community is evident at even coarser levels of taxonomic resolution (e.g., phylum level) at a global scale as well as at finer resolutions at local scales^49^, ^77^, ^78^. Similarly, soil pH was found to be the strongest predictor of fungal OTU richness at the global scale^24^. Soil pH and electrical conductivity (EC) were also found to be key variables describing β‐ diversity variations in bacteria, fungi, archaea, and protist communities in nonforested sites across the western Swiss Alps^79^. A number of explanations have been proposed for soil pH being the major predictor of microbial community structure, i.e., due to variations in the pH preference of different microbes/groups or because the soil pH value is an integrator of a number of soil chemical and physical properties such as nutrient availability, cationic metal solubility/availability, organic C characteristics, and salinity. It has been suggested that soil acidification changes in the assembly and evolution of bacterial communities, that is, assembly of specific bacterial communities through niche‐based exclusion, can be seen at global to local scales and short‐ to long‐term successional trajectories^33^, ^47^, ^80^, ^81^. Bahram et al.^49^ reported that the relative abundances of genes that encode for several metabolic and transport pathways increased, with soil pH contributing to the direct effect on community structure, causing a greater metabolic demand for specific functions under alkaline conditions.

Reports on soil pH effects on temporal variations in microbial community structure are variable^82^, ^83^. Unlike soil pH, salinity and cation exchange capacity were found to be significant drivers of soil microbial community diversity and distribution or relative abundances of different groups, but their predictive capacity of variability can vary at different spatial and temporal scales^13^, ^30^. For example, at the phylum level, *Actinobacteriota* were found to have a niche optimum for salinity of “Very Saline,” whereas the phylum *Verrucomicrobiota* and five of the six subtaxa classes had no defined salinity optimum, with only *Opitutae* showing an optimum of “Moderately Saline”^30^. From a functional group perspective, methane oxidizers were found to show niche partitioning across salinity and moisture gradient in Australian soils and this niche partitioning was suggested to have complex genetic underpinnings^84^, ^85^. Soil Ca concentration was also shown to be a significant predictor of microbial community composition, for example, a strong positive influence on the richness of fungi, especially *Basidiomycota* and ectomycorrhizal fungi, at a global scale^24^, ^49^.

#### Soil moisture and temperature

Soil moisture is one of the important environmental factors influencing microbial community composition and diversity directly through changes in habitat properties and indirectly by affecting the plant biomass production and vegetation composition. The effects of moisture (mean annual precipitation, MAP; rainfall/drought)‐ and temperature (mean annual temperature, MAT)‐related factors are considered more important in studies on both spatial and temporal variations in the composition and diversity of soil microflora, protista, and biological functions^3^, ^13^, ^24^, ^25^, ^39^, ^43^, ^86^, ^87^, ^88^. Their importance in spatial and temporal analyses is considered to be greater in regional and microscale comparisons (Figure 2). The effect of temperature is mostly seen at larger scales both for spatial and temporal variations and depending on whether annual winter freezing occurs.

The association between MAP (surrogate for soil moisture) and MAT with microbial gene functions is considered stronger than that with taxonomic diversity in part due to the responses in specific metabolic functions to climate than local soil functions^49^. Also, differential responses of fungi and bacteria in terms of environmental factors and gene functional diversity may be contrasting, which can be partly attributed to fungi forming hyphal networks, which enable greater tolerance to water limitation. Higher MAP seems to be generally associated with higher fungal diversity at larger spatial scales (e.g., regional and global)^24^, ^49^. Due to the seasonal variations in moisture and temperatures, significant differences in community composition and diversity have been observed at all time scales and spatial levels^89^, ^90^, ^91^, ^92^ (Figure 2). However, Lauber et al.^41^ reported that time of sampling was less important when comparing the β‐diversity of soil bacterial communities in agricultural soils and in successional grassland soils because temporal shifts in bacterial communities may be a product of complex interactions between the soil environment and the more diverse plant community, hence less predictable.

In spite of large seasonal fluctuations in soil temperature and moisture, most of the studies investigating climatic variable‐based responses in microbial communities generally use aggregated values through MAP and MAT estimates for studies at all spatial scales. In view of the well‐known strong and significant responses in the community composition both in terms of taxonomic and functional groups to changes in soil moisture and temperatures, the use of aggregated values likely does not provide a true picture of their influence on microbial communities. Additionally, due to the stronger association of specific metabolic functions with these climate variables, the use of such aggregated values is less likely to provide the true picture of their impact.

#### Toxic chemicals and pesticides

Some soils receive toxic chemicals as wastes from industrial process, spills, waste, or other biosolid disposals, with petroleum products, heavy metals, and solvents being the most problematic. Depending on their concentrations, these can directly affect the soil biota and hence rank as important influencers of the microbial community in affected areas. Application of pesticides (e.g., fungicides and herbicides) is much more widespread and has become an essential management practice in modern cropping systems both in rainfed and in irrigated agriculture as they play an increasingly crucial role in maintaining global food security. Before release for field use, these chemicals are subjected to systematic evaluations for their potential nontarget effects on biota, including responses of microorganisms in terrestrial and aquatic environments, especially when applied at recommended doses. However, there are reports of nontarget changes in soil microbial composition and function from the use of some pesticides (e.g., herbicides, fungicides, and insecticides), in particular from repeated application, application of chemical mixes, and due to the development of resistance^93^, ^94^, ^95^, ^96^. For example, the effects of commonly used herbicide glyphosate were initially considered minimal, with limited consequences to overall microbiome composition and function. In view of its intensive use recently, increasing evidence of microbe and ecosystem effects is being reported^96^.

The effects of pesticides on soil‐ and host‐associated microbiomes can be either direct through changes in microbial survival and growth or indirect through changes in the environment. However, such effects are generally considered to be modest on the overall microbiome, although significant but transient^93^, ^97^. Also, in view of the diverse array of chemicals used in a single crop season, for example, multiple herbicides, fungicides, and insecticides and their toxicities, any significant changes in soil microbial community composition should be considered more important for responses in specific functional groups^93^, ^98^. As most currently registered agrochemicals are degraded through microbial actions, the magnitude and persistence of their effects are influenced by the diversity of microbes before application. Additionally, responses in microbial community composition from agrochemical applications could be due to both increases (e.g., herbicide‐metabolizing microbes) and decreases in the abundance of some specific microbes and/or composition of microbial groups^96^, ^97^. While many of the fungicides recommended for use are suggested to have minimal or no effects on soil fungal communities, as they are selected for controlling specific pathogenic species, significant impacts on overall soil fungal community structure have been reported^93^. Overall, in view of their common usage and differences in microbe sensitivity to the diverse array of chemistries, pesticide use history should be considered as one of the factors in determining the causes of variations in microbial community composition in agricultural systems both spatially and temporally (Figure 2).

### Host‐related factors

Host effects on microbiome composition and diversity can occur through supply of C and nutrients, release of secondary metabolites and signal molecules in the rhizosphere, and influence through dispersal. Host genotype, physiology, and plant compartment also influence the associated microbiomes as the host plant uses a filtering mechanism to select/enrich specific members/groups from the environment^53^. The effects of microorganisms and host interactions inside and outside the host surfaces influence immunological, ecological, and environmental processes. These interactions can affect the host physiology and metabolism and contribute to host adaptation to a changing environment both at individual organism and ecosystem levels. Although the host's internal microbiomes are more likely linked to or modulated by host factors, the external or surface‐associated microbiomes may respond more strongly to changes in edaphic and environmental factors. For example, meta‐analysis of the data set from the Earth Microbiome project and other data indicated that external microbiomes of 654 species including plants, insects, amphibians, nonhuman animals, and humans were most affected by changes in the mean daily temperature range and seasonal precipitation. In contrast, microbiomes inside hosts were most affected by host factors such as phylogeny/immune complexity and trophic level/diet plus climate^99^. However, in a study of soil samples from North and South America, bacterial diversity was unrelated to variables that typically predict plant and animal diversity^6^.

While the biogeography of microbial communities is primarily controlled by edaphic variables, it differs from the biogeography of macroorganisms. Thus, the need for information/metadata on host‐related factors to describe variations in terrestrial microbiome composition and diversity is situation‐dependent for understanding both spatial and temporal variations (Figure 2). For example, due to the variations in seasonality in the presence of and active growth of plant hosts, host‐related information is important to understand the temporal differences in microbiome structure from field to regional scales, and in particular at larger scales (Figure 2).

However, Hayes et al.^100^ observed differences in bacterial and fungal community composition and abundance locally at a plot scale, i.e., between and within plant rows of single and mixed species of grass–legume pastures. Such effects can be attributed to direct rhizosphere‐based effects on microbes through interspecies and intraspecies interactions and indirectly by changing soil habitat conditions^101^. Additionally, plant traits combined with soil abiotic factors were considered to be responsible for variations and patterns in soil microbial communities from local to landscape and regional scales, i.e., plants with conservative strategies support fungal‐dominated communities compared to bacterial‐dominated communities by plants with exploitative strategies^102^, ^103^.

Host type‐based differences in shaping soil microbial communities are also due to variations in the root architecture, rhizodeposition, and growth patterns, for example, variations between legumes versus cereals and grasses. For example, Thion et al.^104^ reported the presence of strong links between ammonia oxidizer community ecology and plant functional traits i.e., rhizosphere of plants with high root N concentrations (high N demand) favored archaeal ammonia oxidizer communities over bacterial ammonia oxidizers. In addition, variations in soil microbial community structure associated with different grass species less than that with legume species were also reported^105^. Similarly, land use‐ and season‐based changes in soil microbial communities in agricultural systems are due to both the presence of a growing plant during a particular part of the season and the type of plant/vegetation^89^, ^92^. Plant species with finer roots and higher resource acquisition traits (e.g., higher shoot N levels) also seem to favor lower diversity of arbuscular mycorrhizal (AM) fungi (AMF) and more diverse putative fungal pathogens, with an overall impact on vegetation dynamics^106^.

One of the well‐studied host–fungal interactions is the plant–mycorrhizal symbioses. This is suggested as a key factor in the belowground microbial network essential for effective functioning of terrestrial ecosystems, especially native and forest vegetation, and host‐based variations in AMF are significant factors^107^. Essentially all species of *Glomeromycota* and some fractions of *Ascomycota* and *Basidiomycota* are known to form mycorrhizal symbiosis with plants. In a worldwide study, the biogeography of AMF showed very low endemism in the AM fungal DNA data for 1014 plant‐root samples and is unexpectedly driven by efficient dispersal, probably via both abiotic and biotic vectors, including humans^108^. Also, the richness of AM fungal communities in individual plant roots or vegetation plots varied in relation to spatial distance and environmental gradients. Since the dispersion of AM fungal species is generally limited to short distances, that is, autochthonous and allochthonous propagules, AM fungal communities are spatially structured and patchily distributed even in homogeneous local environments. Hence, the relative importance of environmental filtering of fungal communities to dispersal is considered as scale dependent and varies depending on plant biomes, soil pH, C/N ratio, phosphorus (P), etc.^109^.

Crop growing seasons and the type of crops grown in each season differ in different agroecological regions of a country/region and globally, for example, winter versus summer crops and multiple crops in a year. It is important to consider this information both in spatial and temporal analyses of variations in microbiome composition and functions especially at larger scales. In agricultural ecosystems, management practices followed for different crops such as land use, season, and fertilizer and agrochemical use can affect soil microbiome community structures both through the direct effects of plant/host type and the indirect effects of management practices including the physical and chemical treatments applied in different crops. Thus, it is important to consider the effects of the host‐related factors when interrogating the variations in microbiome structure both locally and on a regional or global scale.

#### Soil fauna and microbial communities

Protozoa are one of the key trophic functional groups as major consumers (or predators) of bacteria, fungi, and algae with species‐specific effects among protistan communities. Their activity can promote/modulate nutrient cycling processes, soil fertility, and C sequestration^110^, ^111^, ^112^. While the influence of predation by protozoa on microbial activity and functions has been demonstrated widely, its influence on the diversity and composition of bacterial communities is mainly restricted to microsite‐level variations, for example, rhizosphere that is influenced spatially and temporary^113^. Short‐term temporal variations in the activity of protozoa and other fauna due to changes in habitat conditions (e.g., moisture and temperature) directly influence bacterial and fungal communities which is considered more significant at local and microsite levels both spatially and temporally (Table 1). Whereas the effects of larger fauna on microbial composition may be more due to indirect effects through differences in soil type and vegetation‐based variations, hence likely to be more important at larger spatial scales^113^, ^114^. In a recent study across various terrestrial ecosystems across northern and eastern Australia, the bacterial communities (as a food source) and soil invertebrates were the top predictors and responsible for the biogeographic patterns of the protistan consumers^88^. This suggests that the effect of invertebrate fauna was top‐down regulation and a knock‐on effect of trophic cascades. While bacteria and fungi are essential food sources of protozoa, they all also serve as key food sources of predatory nematodes, collembolans and earthworms, thus contributing to trophic cascading effects mostly at larger spatial scales^110^, ^111^, ^114^, ^115^. Macrofaunal effects are also dependent on their composition and abundance as some of them incorporate detritus into the subsurface, thereby increasing the surface area for microbial interactions. For example, the body size range among the soil fauna (grazers and predators) affects microbial communities and soil processes at a range of spatial scales^116^.

#### Animals and manures

The potential effects due to the presence of large animals in agricultural fields and natural ecosystems on soil microbial communities can arise directly from manure and other inputs and indirectly through their effects on plants, for example, growth, plant species richness, changes to moisture, and physical properties of soils, which vary with the grazing intensity and duration^117^. These effects are generally considered more important at larger spatial scales, i.e., field, landscape levels, both spatially and temporally (Figure 2). The application of manure and other organic sources has increasingly been encouraged to reduce the decline in soil organic carbon (SOC) and as a source of plant nutrients in forms that are less readily lost from plant‐available pools. By acting as a source of C and nutrients to soil microbes, application of manures and other such organic sources reshapes soil microbial community composition and alters microbial interactions, in particular specific functional groups and biological functions, depending upon the composition of such inputs^118^, ^119^, ^120^, ^121^. Long‐term application of manures has been shown to increase antibiotic resistance genes (ARGs) as a direct source of ARGs and by increasing the abundance of indigenous ARGs^120^. By adding large concentrations of easily available C and nutrients, these organic inputs can cause significant shifts in microbial abundance and community composition at shorter time scales and through increased plant growth at medium and longer time scales. Additionally, through their impacts on soil habitat structure, for example, improved aggregation, and indirect plant‐based effects, organic input‐based variations should also be considered at longer time scales both spatially and temporally (Figure 2).

### Nutrients

The composition of soil microbial communities including bacteria, archaea, and fungi can be constrained by the availability of nutrient resources, for example, N, P, K, and S, albeit many important biogeochemical processes related to C and nutrient cycles are catalyzed by microbes^57^, ^90^, ^122^, ^123^. Average C and N concentrations of microorganisms are considered to be 44 ± 2% and 12 ± 2%, respectively^122^. The average C/N ratio of soil microorganisms is 6.7, although bacteria generally have lower C/N ratios (ave. 4:1; range 3:1–5:1) than fungi (ave. 10:1; range 4.5:1–15:1)^124^. The total P content in extracted cells from soil ranges from 2.1 to 8.9 fg/cell and the concentration varies based on P availability and P concentrations. Pure culture‐grown bacteria have higher P concentration (18–23 mg/g) than fungi (ave. 9.5 mg/g)^125^, ^126^. While several global to local studies indicate some level of influence of nutrient concentrations on soil microbial communities, variations in nutrient concentrations or availability have a lower impact on the top‐level assembly of microbial communities^25^, ^48^, ^49^. Additionally, the relative importance of various nutrients differs for total microbial community composition, composition and abundance of specific taxonomic (e.g., bacteria, fungi, different phyla/orders) and metabolic and functional groups (e.g., nitrifiers, N‐fixing microbes, S‐oxidizing microbes), and therefore is reported in correlation‐based investigations as having limited impact on soil microbial community composition. The influence of nutrients may also be reflected through concentrations of individual nutrients or their ratios with C or other nutrients (e.g., C/N and N/P/S ratios).

It is well established that physico‐chemical properties such as pH, texture, and other properties like variable charge surfaces can have a significant impact on the concentrations and availability of different nutrients in soils^127^. Furthermore, environmental variables like soil moisture and temperature also have significant impacts on the concentrations of available nutrients, in particular temporally. As a multitude of interactions can impact nutrient concentrations and availability temporally, the proportion of variation in microbial communities that can be directly attributed to nutrients is generally considered to be lower, in particular for overall community composition (Table 1 and Figure 2). Also, methods for nutrient‐related measures not only vary for the different forms of the same nutrient (e.g., total concentrations or different forms) but are also not favored because of methodological complexities (e.g., NH_4_^+^, NO_3_^−^, available P, and micronutrients).

Overall, the dynamic nature of available forms of nutrients influenced by edaphic, environmental, and host‐related factors suggests that they should be considered for both spatial and temporal variations in soil microbial communities and at different scales, especially for investigations of specific functional or taxonomic groups and individual genera (Figure 2). Additionally, due to use of inappropriate measures for nutrients that are highly dynamic, true information on their impact on changes/differences in microbial community may not be obtained. For example, the total N concentration is the most commonly used measure for soil N status, which does not show appreciable temporal and management‐induced changes compared to mineral N concentrations.

#### Nitrogen

N is one of the key elements for microbial growth for almost all groups of microorganisms; hence, soil properties related to N concentration and availability have been found to be significantly correlated with the abundance, composition, activity, and functional capacity of microbial communities in soils both globally and locally. For example, the total N level in a soil does not reflect its bioavailability to microorganisms, i.e., short‐term temporal variations potentially affecting microbial growth and composition. Measures that indicate the level of available nutrient status and C/N ratio were shown to be strongly correlated with the diversity and composition of bacterial and fungal communities independently and fungal to bacterial ratios^48^, ^128^. The positive relationship between soil C:N ratios and fungal: bacterial ratios has been attributed to multiple factors such as (i) direct influence on the relative abundance of bacteria due to their requirement for more N per unit biomass C than fungi, i.e., stoichiometric constraints, (ii) differential antibiotic production abilities of bacteria and fungi in constrained environments, and/or (iii) the integrated effects of many other soil habitat characteristics including the quality of C and fertilizer inputs. For example, an increased production of antibiotic compounds by fungi under high C/N environments and broader activities of fungi than bacteria in using complex C substrates are suggested to affect fungal:bacterial ratios^49^. Soil C/N ratio has been suggested as one of the best predictors of richness for fungal functional genes and increased antibiotic production of fungi^24^. A decline in the diversity of plant pathogens with increasing C:N ratios has also been reported^24^, ^49^. N levels in soils are also affected by N inputs through anthropogenic activities and N fertilization in agricultural systems.

The effects of N availability on bacterial and fungal communities, in particular N cycling communities such as diazotrophs and denitrifying organisms, can be seen both spatially and temporally and at global and local scales^43^, ^129^ (Figure 2). For example, it is important to consider the influence of temporal variability in available N levels on the composition of various functional groups, phylogenetic and/or functional, to better understand the dynamics of microbiome response to environmental (e.g., moisture and temperature) and land management practices that may be more important for local scales. However, at regional and global scales, N‐related properties could also influence variations in vegetation/plant species composition as elevated N levels are known to support more r‐selected plant species. Results from comparative metagenomic, phylogenetic, and physiological analyses across varying N availability scenarios suggest that N fertilization can cause significant phylogenetic shifts in bacterial and fungal communities; for example, increased relative abundance in copiotrophic taxa alters catabolic capabilities, and increased relative abundances of genes associated with protein metabolism, and DNA/RNA replication result in an overall shift in microbial life‐history strategies^13^, ^128^. The effects of N availability including N fertilizer application on the diazotrophic microbes and organisms harboring genes involved in denitrification and other N cycling processes also contribute to responses in microbial community composition phylogenetically and functionally, making the type of N metric measured a key component of soil habitat characterization.

#### Phosphorus

Microorganisms play an integral role in the P cycle in soils, i.e., specific groups of microbes involved in the mineralization/release of P from sparingly available forms of inorganic and organic P through production of exoenzymes, induction of metabolic processes including efflux of protons and organic anions, and alteration of sorption equilibria resulting in transfer of orthophosphate ions into soil solution and microbial turnover of organic P^130^, ^131^. However, acquisition of sufficient P for supporting essential cellular processes and energy functions such as DNA synthesis and repair may be difficult for microbes in highly weathered and tropic soils, calcareous soils in the Mediterranean regions, etc. Thus, P limitation in soils can play an important role in shaping the composition of soil microbial communities and their metabolic capabilities in bulk soil and rhizosphere environments, i.e., differences in P availability influencing soil bacterial communities and their functional attributes^132^. Available P levels explained a significant percentage of the variation in overall soil bacterial and archaeal community composition in the Australian and Panamanian soil data and fungal community composition in subtropic forest systems, highlighting the broad importance of P availability in shaping belowground communities across diverse ecosystems and soil types^121^, ^132^, ^133^.

Soil P level was also found to be one of the key factors driving the microbiome structure in the rhizosphere of maize genotypes, for example fast‐growing taxa more enriched in high‐P soils and vice versa^134^. Also, P fertilization can significantly impact bacterial and fungal community composition in oxisol and calcarosol soils^134^, ^135^. P availability has been found to significantly influence AMF abundance, plant colonization by AMF, and abundance and composition of P‐solubilizing bacteria^109^, ^136^, ^137^. The influence of P availability on microbial composition is mostly relevant in spatial analyses at field, regional, and global scales, as P availability is regulated by soil pH, physical and chemical properties including minerology, availability of C, concentrations of Ca, and carbonates, etc. (Figure 2). For example, P deficiency for plant needs in calcareous soils and weathered and tropical soils is considered to be linked to variations in microbial community composition^138^. Microorganisms living in some of these environments have mechanisms of adaptation to phosphate deficiency and excess i.e., catabolism of phosphonates is one of the mechanisms used by bacteria to scavenge phosphate in P‐limited soils, whereas significantly higher microbial potential for utilization of inorganic P was found in high‐P soils^132^. Also, in low‐P soils, microbial taxa responsive to extractable soil P levels are more likely to have traits typical of oligotrophic life‐history strategies. For example, temporal variation in rhizosphere microbial composition is a product of plant‐induced P depletion and changes in bacterial and fungal composition. This can be a result of selective enrichment of microbial species or groups with some plant species having specific traits related to the transformation of inorganic and organic P forms into bioavailable pools^130^.

#### Micronutrients

The availability of S and micronutrients such as Zn, Cu, and Mo can influence microbiome composition and functionality in particular microbial groups through direct nutritional‐based effects on all or specific members of the microbial community or via indirect effects through plant‐based variations, i.e., nutrient responses to plant growth resulting in changes in the vegetation composition and subsequent rhizosphere influences. However, since microbes often have active mineral uptake and do not need many of these elements, they are not generally considered to exert a significant influence on the overall soil microbiome composition, except in unusual geochemical conditions. Additionally, their influence may be masked by the stronger effects of factors that have more critical attributes (e.g., pH and organic C). The ranking shown in Figure 2 considered reports that show information for either direct or indirect effects of micronutrient availability on total microbial communities or specific functional groups.

Some examples where micronutrient levels may or can cause enrichment or depletion of specific microbial groups include microbes involved in S transformations, nitrifying organisms, and N_2_‐fixers^139^, ^140^, ^141^. S is an essential component of a number of biomolecules including proteins, vitamins, hormones, and lipids for the generation and conservation of biochemical energy^142^. S availability is also crucial for the biosynthesis of nitrogenase in diazotrophs. Different bacterial and fungal species/groups are involved in specific S transformations; hence, availability of various S compounds may vary in their effects on the abundance of specific functional groups. Addition of elemental S can influence the relative proportions of bacterial phyla and bacteria involved in S oxidation and other nutrient‐mobilizing traits in rice, wheat, and soybean fields^141^, ^143^, ^144^, ^145^. In rice fields, added elemental S resulted in an increased ratio of *Acidobacteriota* and *Bacteroidota* to *Planctomycetota* and *Chloroflexota* ^145^. High concentrations of S in soils can also be considered a pollutant and can impact microbial composition by affecting the physico‐chemical properties of soils, especially reducing soil pH^142^.

The presence of many heavy metals (e.g., Cu, Ni, Mo, Fe, and B) can influence nitrifying bacteria both as stimulants under deficient conditions and by exerting toxic effects when concentrations are high. For example, Cu is an essential co‐factor in the enzyme involved in nitrification; hence, limited Cu bioavailability can constrain bacterial and archaeal nitrifiers, whereas higher concentrations of Cu can have negative effects on the abundance and composition of nitrifying organisms^146^. The bioavailability of micronutrients such as Zn and Mo is influenced by specific microbes (e.g., Zn‐mobilizing bacteria^147^) and, at the same time, their availability and long‐term exposure to high concentrations can influence the overall microbiome composition^148^. For example, Mo‐Fe protein is an important component of the nitrogenase enzyme in diazotrophs, and thus limited availability of these elements can influence their abundance and functionality, and Mo fertilizers can increase their abundance and functionality^149^. Similar effects were also observed for boron fertilizer application, which led to increases in nitrification and N fixation in soybean rhizosphere in subtropical alluvial soils^149^.

## STOCHASTIC‐ VERSUS DETERMINISTIC‐BASED COMMUNITY STRUCTURE

It is quite common to obtain poor correlations between soil microbial community composition and the measured environmental drivers; for example, values of 25% or less are often correlated to environmental factors, which provide weak determination of the drivers. This has led to consideration of what is not being measured and can be measured, what are the appropriate scales of assessment, and more recently, whether the outcome is truly random, i.e., stochastic. If it is the latter, what are the stochastic vs. deterministic features, which ones are important, and how can they be conceptualized and even qualified? Over the last decade, several authors have provided guidance about soil microbial community assembly, often drawing from macroecology principles^79^, ^150^, ^151^, ^152^, ^153^, ^154^, ^155^. While both stochastic and deterministic processes often jointly influence microbial community composition, the relative contributions of specific communities (e.g., denitrifiers, diazotrophs, bacteria vs. archaea, and fungi) and their processes are likely scale dependent^45^, ^129^, ^156^, ^157^, ^158^.

Zhou and Ning^10^ reviewed the ecological basis for stochastic vs. deterministic features and evaluated them to gain an understanding of microbial community assembly. The deterministic processes are environmental filtering (selection) and biotic interactions (competition, predations, and mutualisms), and of lesser significance, nonrandom dispersal (active propulsion) and nonrandom diversification (positive mutations). The stochastic processes are: historical (priority effects), dispersal limitations, ecological drift, birth, and death. For the soil environment, historical effects and dispersal limitations are probably major factors causing poor correlations with soil determinants, along with a large proportion of the community being dormant or physiologically unresponsive or nearly so to current conditions. Conceptually, it is easy to understand that not every ecotype is in every gram of soil from the studied condition, or if it is present, it is not feasible to detect in large volumes of soil, for example, 1 kg or more. These community assembly principles are derived from macro‐ecology, where species is the unit of selection for ecology and evolution. This is not necessarily the case for prokaryotes, and it is also not easy to measure prokaryotic “species”.

**Figure 3:**
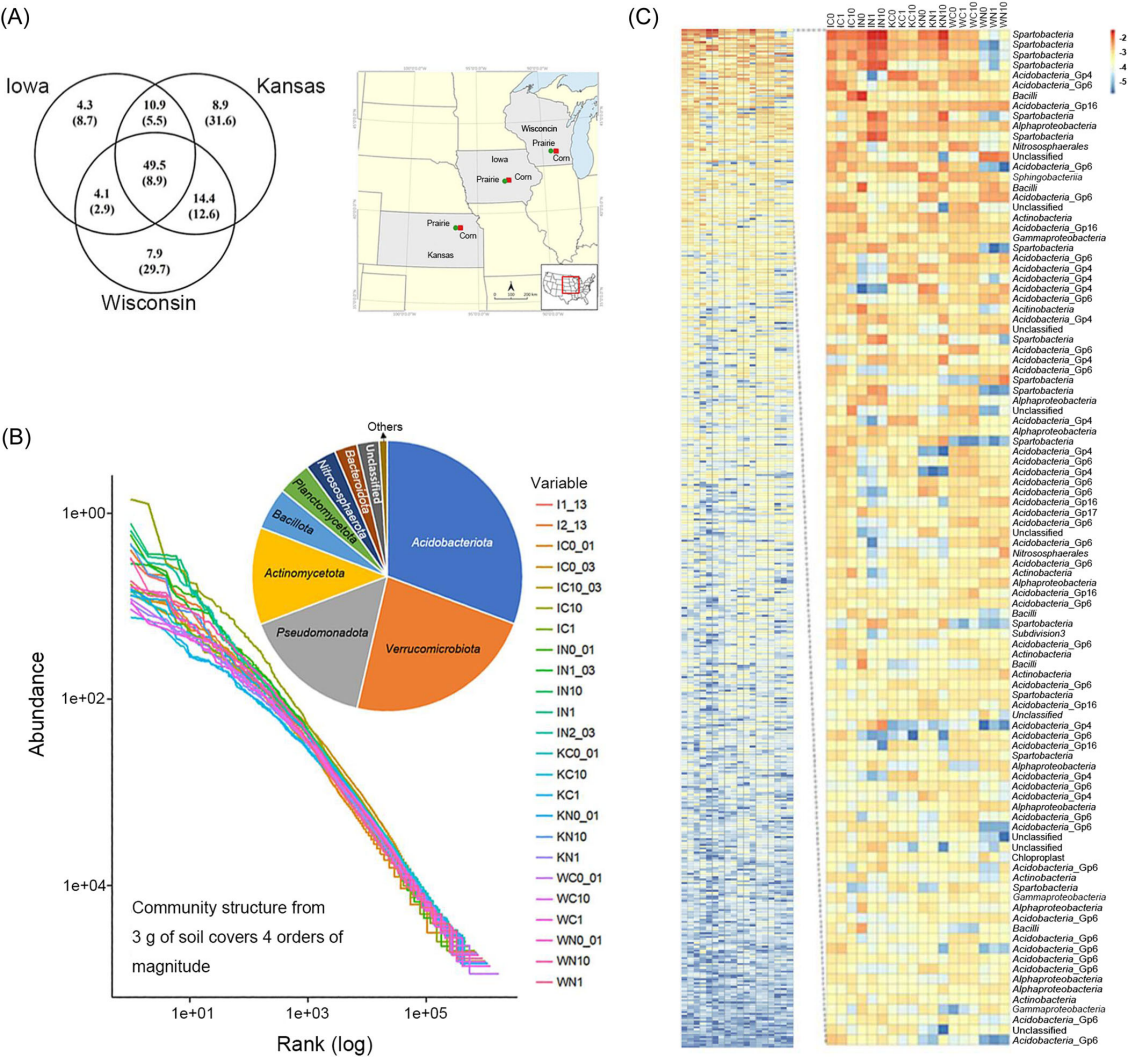
Commonality of USA Midwest bacterial populations in soils from native tallgrass prairie fields (green ball) and that experienced >100 years of cultivated agriculture (Corn, in red square) in a transect from Kansas, Iowa, and Wisconsin. (A) Venn diagram with values for relative abundances of common phylotypes in each soil and percentage of common phylotypes across soils with % values in parenthesis. (B) Rank abundance of 16S rRNA phylotypes at log–log scale and a pie chart showing relative abundances of major phyla. (C) Heat map showing similar declining density of 529 common phylotypes in the 18 soil samples randomly selected 360 K reads from approximately averaging 1 M reads per sample (unpublished data from W‐J Sul and JM Tiedje). Surface 0–10 cm soils collected during 2009 from native prairie fields and nearby long‐term agricultural sites matched in soil type, texture, slope, aspect, and drainage were analyzed for bacterial community composition using the 16S rRNA amplicon (V4 region). Further details of the sites, sampling, and other detailed genomic analyses can be seen in Ref.^160^.

There are other factors beyond these core assembly principles that limit the assessment of drivers of the soil community assembly; the physical and biological scales evaluated. In short, evaluation of how much of the community is deterministic vs. stochastic is “context‐dependent”, i.e. (i) what is the spatial scale (the larger the spatial scale, the more stochastic the outcome)? (ii) what is the temporal scale of the driver effect (weeks to decades or more), (iii) what is the taxonomic scale (genotype, species, and genera—e.g., rRNA or higher taxonomic levels)? and (iv) what are the physiological features of the organism that affect its longevity (spore former, resilient populations, or poor survivors)? Another complication is how proximate is the environmental feature that is measured in terms of the organism's selection traits or its process? In the example of denitrifiers mentioned above (Figure 1), the primary growth and activity determents are well known, but what is measured may be distal to the organism and hence its effect is muted and, for some determinants, contradictory in different soil sites. Given these many biological and environmental features and measurement challenges, it is understandable why it is difficult to reliably link soil communities with their drivers. That said, at large spatial and biological scales (e.g., phyla and class), soil microbiomes do correspond to their soil ecosystem type, cf. Sul et al.^159^, and within the same ecosystem type; for example, US Midwest prairie mollisols have the same taxa at the genus scale (Figure 3A) and similar rank order of dominance (Figure 3B), but whether the soil was native prairie or 100 years of cultivated agriculture did not alter the microbiome at this taxonomic level, reflecting a stable community. This shows that time—100 years—was not sufficient to show differences due to cultivation. However, at a finer level of taxonomic and genetic resolution, differences were seen due to cultivation and due to location in the 500 km transect^160^. This study also shows that the dominant taxa, for example, *Acidobacteriota* and *Spartobacteria* (Figure 3C), which are persistence specialists, would probably not be expected to respond at the 100‐year time scale, hence muting community‐wide sensitivity to cultivation.

## SUMMARY

Soil microbial communities are exceptionally complex due to the spatial heterogeneity in physical and chemical properties and the highly protective nature of soil habitat coupled with a diverse array of environmental conditions and management practices to which they are exposed. Significant portions of the soil microbial community structure are likely stable, i.e., only very slowly responsive to varying environmental conditions, except when new organic carbon enters their space. Previous discussion on the variability in microbial community in terrestrial ecosystems is generally based on correlations between community diversity and composition and the soil abiotic and biotic (aboveground/host) factors, with limited explanation of the mechanistic factors that can influence microbial growth and function. In general, biologists have accepted that fundamental RoL that constrains microbiology do exist and apply across scales from genes to ecosystems^161^. However, for advancing the study of RoL, it is important to identify the hierarchical structure of the subordinate rules and their context dependency^162^. Also, the usefulness and interoperability of the extensive microbiome data (which are being collected and boosted by the interdisciplinary microbiome research worldwide) beyond their original location of the study, for identifying changes to community structure and trait responses, are dependent upon appropriate metadata collection.

This concept paper briefly discusses what is known from the literature but more importantly organizes this information based on the first principles of what is essential for microbial growth in terms of those that (i) supply energy (organic C and electron acceptors), (ii) major environmental effectors or stressors that often limit growth (e.g., pH, salt, drought, and toxic chemicals), (iii) influences of macroorganisms, and (iv) nutrients in order, N, P, and other micronutrients and metals, or physicochemical influences of their soil “home”. The relative contributions of specific microbial communities and processes are also scale‐dependent both spatially and temporally. In short, the soil microbial community composition is jointly influenced by both stochastic and deterministic processes that are scale and context‐dependent.
